# You've Been Framed: The Impact of Risk and Time Framings on Contraceptive Preferences in a Discrete Choice Experiment

**DOI:** 10.1002/hec.70039

**Published:** 2025-09-17

**Authors:** Matthew Quaife, Giulia Chiandet

**Affiliations:** ^1^ Patient‐Centered Research Evidera London UK; ^2^ London School of Hygiene and Tropical Medicine London UK

**Keywords:** contraception, discrete choice experiment, risk framing, time framing

## Abstract

Previous research shows that choices are influenced by how probabilities are presented, that we value losses more than gains, and that we misunderstand cumulative probabilities over time. These factors are important when designing discrete choice experiments (DCEs) because almost all include some representation of probability over a time period. Contraceptive choice is one of the most common health choices and requires people to trade‐off between efficacy, side effects, and modality. We used a DCE to explore whether people chose differently when faced with positive or negative framings of contraceptive effectiveness or valued 1‐year or 3‐year cumulative risks differentially. We developed a simple eight‐task DCE with three attributes: effectiveness, administration frequency, and (non‐)hormonal nature. Participants saw effectiveness as either positively or negatively, and with numerically equivalent 1‐year and cumulative 3‐year effectiveness values. We used mixed multinomial logistic regression models with interaction terms and explored preference heterogeneity. The negative frame increased sensitivity to effectiveness by 18% (*p* = 0.04) and sensitivity to cumulative effectiveness over 3 years was 10% less than over 1 year (*p* = 0.01). Preferences were heterogenous with respect to attributes but not framing effects. Attribute framing substantially affected preferences for effectiveness, and decisions around risk presentation should be reported transparently.

## 1 Introduction

Contraceptives are one of the most commonly used health technologies, used by 76% of women of reproductive age in the UK (French et al. 2020). Despite this high usage, it has been estimated that 16% of pregnancies in the UK are unintended (Wellings et al. 2013), and a recent survey from Scotland found that around 96% of respondents reported some dissatisfaction with contraceptive provision (Cheney et al. 2019). Many different methods are available and, until recently, potential users accessed contraception almost exclusively through consultation with trained healthcare providers. Yet contraceptives are increasingly advertised and available through non‐traditional channels including social media, where fertility awareness contraceptive apps such as Natural Cycles advertise direct to consumers (Natural Cycles 2020). Such marketing relies in part on describing contraceptive effectiveness, which shows probabilistic effectiveness information directly to potential users without a healthcare provider intermediary.

The effectiveness of a contraceptive method is the inverse of its failure risk. It is typically presented as an effectiveness percentage over 1 year (nhs.uk 2017; Planned Parenthood 2020). This way of describing effectiveness can be considered a positive framing, and the failure rate a negative framing. We know that losses and gains are not valued symmetrically (Kahneman and Tversky 1979), and empirical studies have found that people attribute more utility to a loss than an equivalent gain (Tversky and Kahneman 1991; Neuman and Neuman 2007; Veldwijk et al. 2016). Despite effectiveness being a primary consideration when choosing a contraceptive method (Wyatt et al. 2014; Trussell 1991), to date such framing effects have not been explored in the context of contraceptive choices.

Discrete choice experiments (DCEs) are a quantitative stated preference method, applied to a wide range of health economic questions (Clark et al. 2014). Previous DCEs exploring contraceptive choices demonstrated that women preferred contraceptives with fewer side effects, higher efficacy in preventing pregnancy, and less frequent administration (Weisberg et al. 2013; Knox et al. 2012; Minnis et al. 2019; Mueller et al. 2018; Agyei‐Baffour et al. 2015; Fiebig et al. 2011). More broadly, research has shown that probabilities are not always understood well, and individuals' risk perceptions may be influenced by behavioral biases and framing effects (Visschers et al. 2009; Lipkus 2007; Peters et al. 2011; Berry 2006). Outside of contraception, there is mixed evidence of whether choices in DCEs are affected by risk presentation (Veldwijk et al. 2016; Wright et al. 2019; Nickel et al. 2018; Gong et al. 2017; Grisolia et al. 2018; Vass et al. 2014; Quaife, Vickerman, et al. 2018; Jiang and Fraenkel 2017). As a recent review highlights, there is a large variety in the way risk is presented in DCE studies and many authors do not explicitly justify their choice of presentation (Fernandez et al. 2025).

The time period over which contraceptive products are effective varies (e.g. a daily pill vs. a 4‐year intrauterine device), yet contraceptive effectiveness is almost always presented for 1 year. An informal literature review found no examples of DCEs which directly explored whether respondents value cumulative risk in a consistent way when presented over different time periods. A systematic review of DCEs found that only half of studies explicitly stated the time frame over which risks might occur (Harrison et al. 2014). Outside of DCEs, evidence suggests that people may not accurately consider the additive nature of risk over time (Visschers et al. 2009), and that the extent cumulative risks are considered accurately is correlated with individual characteristics, for example education (Fuller et al. 2004; Knäuper et al. 2005; Keller et al. 2006; Doyle 1997). One study looked at how individuals estimated cumulative risk for contraceptives (Shaklee and Fischhoff 1990), and found that many did not consider that the risk of contraceptive failure accumulates over time, whilst those that did tended to underestimate the likelihood of pregnancy for longer time periods. Even if people value cumulative risks accurately, under a discounted utility model we expect individuals to value benefits that are temporally closer more than those further in the future (Hess et al. 2005). The National Institute for Health and Care Excellence (NICE) guidelines state that health benefits and costs should be discounted at a rate of 3.5% a year (National Institute for Health and Care Excellence 2001).

This study developed a simple, stylized DCE with the intention of exploring how the presentation of effectiveness and time attributes affected preferences, among a convenience sample of women recruited through UK‐based university research groups and social media platforms. We explored if contraceptive effectiveness was valued differently with positive or negative framings; and how respondents assessed cumulative risk over different time periods.

## 2 Materials and Methods

### 2.1 Sampling and Survey Structure

Data were collected between 23rd June 2020 and 2nd August 2020 using the OnlineSurveys platform. A convenience sample was recruited through advertisements on several survey sharing and birth control discussion Facebook groups, university research groups, and on *socialpsychology. org*. Due to the primary interest to explore framing effects, rather than make statements around population preferences, the sample was not intended to be representative of the general population but sought to reach a sample of women likely to currently be making contraceptive choices. Inclusion criteria were women aged 18–45, who were able to read and understand English, and who were currently using of at least one form of contraception. No compensation was provided to participants.

Prior to the DCE the participants completed a short screener. After the DCE, participants were asked to rank the contraceptive attributes in order of importance. Finally, a section elicited socio‐demographic characteristics by which preferences were hypothesized a priori to potentially vary: education level, risk‐attitudes, experiences of unplanned pregnancies, and current pregnancy aversion.

A behaviorally validated risk aversion scale was asked ranging from 1 to 10, with 1 being “not at all prepared to take risks” and 10 being “very much prepared to take risks”, as used in the German Socio‐Economic Panel Study (Dohmen et al. 2011), and shown to perform better than other risk aversion measures, such as the Holt and Laury risk titrator (Holt and Laury 2002), in predicting risky health behaviors (Szrek et al. 2012). Attitudes toward becoming pregnant were recorded using a scale that from 1 to 10, with 1 being “worst feeling imaginable” and 10 being “happiest you could feel”. These were transformed into binary variables based on a cut off at the 50^th^ percentile.

### 2.2 DCE Design

Because the primary intention of the DCE was to explore framing effects, the design was simple and sought to include two attributes in addition to contraceptive efficacy. Attributes were chosen based on empirical studies of contraceptive choices (Weisberg et al. 2013; Knox et al. 2012; Mueller et al. 2018; Fiebig et al. 2011), which found that effectiveness, frequency of administration, and hormonal/non‐hormonal mechanism were important and distinct determinants of contraceptive choice. Effectiveness and administration frequency levels were chosen based on existing methods provided in the UK; long‐acting reversible contraceptives were omitted due to their greater‐than 99% effectiveness (nhs.uk 2017), requiring different presentation than simple percentage to ensure participants understood higher effectiveness, and concerns of potentially dominating decision making as effectiveness approached 100%. Final attributes included in the DCE and the wording of the framings are shown in Table 1.

**TABLE 1:** DCE attributes and levels.

	Level
Attribute	
Effectiveness	99%, 95%, 90%, 80% (effectiveness over 1 year) 97%, 86%, 73%, 51% (corresponding effectiveness over 3 years)
Frequency of administration	One per day, one per month, one every 3 months
Type of contraception	Hormonal, non‐hormonal
Risk framings	
Positive vs. negative frame: Between‐subjects	Positive framing: 95% of women will not become pregnant in 1 year Negative framing: 5% of women will become pregnant in 1 year
Yearly vs. cumulative risk frame: Within‐subjects	One year frame: 95% of women will not become pregnant in 1 year Three‐year frame: 86%* of women will not become pregnant in 3 years *Where 95%^3^ = 86%

Three‐year equivalents of the yearly effectiveness were calculated simply as *1‐year effectiveness^3^.* In reality, this may over‐overestimate 3‐year protection as it assumes constant fertility over time and across individuals who do and do not become pregnant, however allows numerical equivalence across time periods.

**FIGURE 1:**
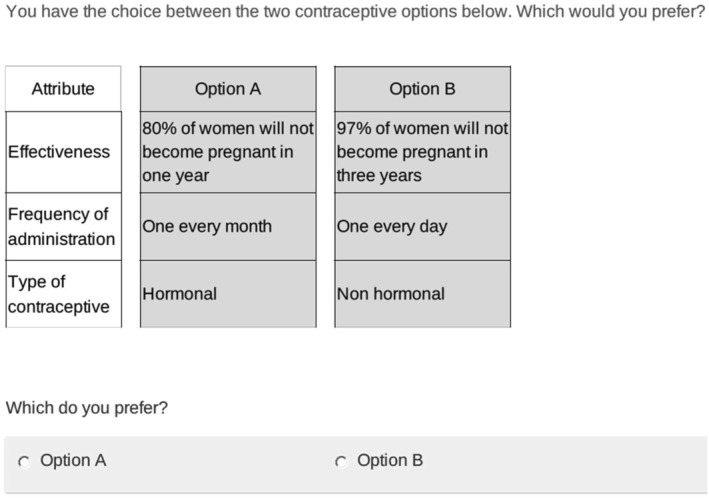
Example choice task from survey platform.

The DCE was unlabeled and presented participants with a forced binary choice between two options, as shown in Figure 1. An opt‐out was not included as all the women included in the study were currently using at least one contraceptive method, and we did not seek to estimate unconditional demand (Veldwijk et al. 2014). The DCE was piloted using a 12‐task fractional factorial design generated in NGENE software (ChoiceMetrics. NGENE). A pilot questionnaire was conducted on a convenience sample of 11 respondents and included phone‐based “think aloud” tasks as participants completed the questionnaire to assess tool clarity and comprehension. The wording of frequency levels was changed following the pilot, from “three‐monthly” which was misunderstood as being “three times a month”, to “one every 3 months”. Data from the pilot were used to estimate main and framing effects priors and these were used to generate an 8‐task D‐efficient design using NGENE (ChoiceMetrics 2012); pilot data were not included in final analyses.

### 2.3 Randomization and Framing

The positive versus negative risk framing was varied between‐subjects, where half of the sample saw one version and the other half, the other. The OnlineSurveys tool did not support randomization, so participants were randomised based on their age where even number aged respondents saw the positive framing, and odd number aged respondents the negative framing. The cumulative framing, that is the effectiveness in 1 year versus the effectiveness over 3 years, was varied within‐subjects in the experimental design, and included in the pilot and final DCE designs. Including variations in both within‐participants would have provided maximal experimental power in exploring framing effects, but also required maximal cognitive burden from participants seeing presentation changes in two attributes; hence participants saw a consistent risk framing across all tasks.

#### 2.3.1 Descriptive Statistics

We summarized characteristics of the whole sample and framing arms. *T*‐tests and Chi‐square tests tested for differences between the two groups. Participants' direct attribute rankings were summarized, and Chi‐square tests used to test for differences between the rankings of attributes in the positive and negative framing.

#### 2.3.2 Discrete Choice Analyses

Choice data were analyzed using mixed multinomial logit (MMNL) models, which relax restrictive assumptions in simpler choice models and account for unobserved taste heterogeneity (Quaife, Eakle, et al. 2018; Hess et al. 2005; Hole 2008). In a random utility framework, the utility $U_{ni}$ derived by individual *n* from alternative *i* is given by an observable element $V_{ni}$_,_ a random component $\Gamma_{ni}$ characterized by an analyst‐chosen distribution, and an unobservable, random element $\varepsilon_{ni}$:

(1) $$U_{ni}=V_{ni}+\left[\Gamma_{ni}+\varepsilon_{ni}\right]$$

To allow for uncertainty in distribution of their variance, $\Gamma_{ni}$ parameters were assumed random and normally distributed, and 1000 Halton draws used in estimation. The main effects specification of the observable element, $V_{ni}$ is described by Equations (2) and (3):

(2) $$V_{ni}=X_{ni}\beta +\varepsilon_{ni}$$

(3) $$X_{ni}\beta_{i}=\beta_{0}+\beta_{1}{\text{Effectiveness}}_{i}+\beta_{2}{\text{Monthly}}_{i}+\beta_{3}{3\text{monthly}}_{i}+\beta_{3}{\text{Nonhormonal}}_{i}$$

Where ${\text{Effect}}_{i}$, ${Freq}_{i}$ and ${\text{Type}}_{i}$ are attribute levels, and $\beta_{0}$ a constant.

To test for framing effects, a variable was added to the model interacting a binary framing variable with effectiveness. Because framings only affected how effectiveness was displayed, framing parameters were interacted with effectiveness only.

We calculate the marginal rate of substitution (MRS) to assess the amount of effectiveness participants were willing to trade in return for, or to avoid, other attributes. MRS’ are estimated by re‐arranging the utility function Equation (3) by taking the ratio between the marginal utility of one attribute *k* and the marginal utility of effectiveness as follows:

(4) MRSk=∂v∂xk−∂v/∂[Effect]=βˆk−βˆ[Effect]

#### 2.3.3 Preference Heterogeneity

Observable preference heterogeneity was explored including interaction terms in main effects and interaction effects models. Participant characteristics were decided before any models were run, all are reported, and were: age, type of contraceptive currently being used, scores on risk aversion and pregnancy attitude scales, reason for using contraceptives, past pregnancy, and intention to conceive prior to the past pregnancy.

## 3 Results

### 3.1 Participant Characteristics

Table 2 presents the characteristics of survey respondents. In total 752 people participated, with a mean age of 26.7 years. There were no notable differences in characteristics between the participants in the two framing conditions. Overall, 55% of participants saw positively framed DCE tasks and 45% negatively framed tasks.

**TABLE 2:** Participant characteristics.

Characteristic		Total sample (*N* = 752)	Positive framing (*N* = 411)	Negative framing (*N* = 341)	Difference between framing arms *p*‐value
Age: Mean (SD)		26.74 (5.16)	26.77 (4.88)	26.71 (5.49)	0.89^a^
Education, *N* (%)	Primary school	4 (0.53)	2 (0.49)	2 (0.59)	0.48^b^
	Secondary school	16 (2.13)	8 (1.95)	8 (2.35)	
	Higher or further education	91 (12.10)	53 (12.90)	38 (11.14)	
	College or university	374 (49.73)	216 (52.55)	158 (46.33)	
	Post‐graduate degree	247 (32.85)	123 (29.93)	124 (36.36)	
	Doctoral degree	15 (1.99)	7 (1.70)	8 (2.35)	
	Prefer not to say	5 (0.66)	2 (0.49)	3 (0.88)	
Current contraceptive used, *N* (%)	Pill	240 (31.91)	137 (30.21)	103 (30.21)	0.19^b^
	Condoms	115 (15.29)	61 (14.84)	54 (15.84)	
	Non‐hormonal coil or IUD	99 (13.16)	50 (12.17)	49 (14.37)	
	Hormonal coil or IUS	82 (10.90)	54 (13.14)	28 (8.21)	
	Implant	53 (7.05)	26 (6.33)	27 (7.92)	
	Abstinence	52 (6.91)	32 (7.79)	20 (5.87)	
	Withdrawal	29 (3.86)	10 (2.43)	19 (5.57)	
	Natural family planning	28 (3.72)	14 (3.41)	14 (4.11)	
	Vaginal ring	19 (2.53)	9 (2.19)	10 (2.93)	
	Sterilization	16 (2.13)	9 (2.19)	7 (2.05)	
	Patch	11 (1.46)	7 (1.70)	4 (1.17)	
	Injection	7 (0.93)	2 (0.49)	5 (1.47)	
	Emergency contraception	1 (0.13)	0 (0)	1 (0.29)	
Risk aversion, Mean (SD)		3.01 (2.28)	2.96 (2.27)	3.08 (2.30)	0.48^a^
Past pregnancy, *N* (%)	Yes	144 (19.15)	74 (18.00)	70 (20.53)	0.54^b^
	No	603 (80.19)	335 (81.51)	268 (78.59)	
	Prefer not to say	5 (0.66)	2 (0.49)	3 (0.88)	
Pregnancy intention, *N* (%) *Of those previously pregnant*	Intended to	58 (40.28)	28 (37.84)	30 (42.86)	0.66^b^
	Intentions kept changing	7 (4.86)	3 (4.05)	4 (5.71)	
	Did not intend to	78 (54.17)	43 (58.11)	35 (50.00)	
	Prefer not to say	1 (0.69)	0 (0)	1 (1.43)	
Pregnancy aversion *, mean (SD)		1.77 (2.05)	1.73 (2.05)	1.81 (2.06)	0.62^a^
Reason for using BC^c^, N (%)	Avoid pregnancy	709 (94.28)	386 (93.92)	323 (94.72)	0.64^b^
	Make periods lighter	224 (29.79)	125 (30.41)	99 (29.03)	0.68^b^
	Reduce period pain	218 (28.99)	121 (29.44)	97 (28.45)	0.77^b^
	Control period timing	213 (28.32)	118 (28.71)	95 (27.86)	0.80^b^
	Reduce PMS symptoms	116 (15.43)	58 (14.11)	58 (17.01)	0.27^b^
	Reduce acne	95 (12.63)	50 (12.17)	45 (13.20)	0.67^b^
	Other	59 (7.85)	34 (8.27)	25 (7.33)	0.63^b^

*Note:* % of total sample selecting each option.

^a^ *p*‐value from *t*‐tests.

^b^ *p*‐value from χ^2^ or Fisher's exact tests.

^c^ Multiple choice question.

Nearly half of the sample (45%) reported currently using a non‐hormonal contraceptive method. The sample was well educated, with 75% reporting a university degree or higher, and nearly all reported contraceptive use primarily to avoid pregnancy (94%). Four‐fifths had never been pregnant (80%), and among those who had previously been pregnant, over half had not intended to get pregnant (54%).

Participants scoring 1 or 2 on the risk aversion scale (66%) were defined as less risk averse (a score of 1 being “not at all prepared to take risks”). Participants scoring 1 or 2 on the pregnancy aversion scale (57%) were defined as pregnancy averse (as score of 1 being “worst feeling imaginable”). High levels of pregnancy aversion were expected due to the inclusion criteria of at least one contraceptive method.

### 3.2 Attribute Ranking

Two‐thirds of respondents rated effectiveness as the most important attribute (68%), followed by hormonal or non‐hormonal action (23%) and frequency of administration (8%) (Supporting Inormation S1: Appendix Table 1). At the 10% level, there were no significant differences in attributes rankings between participants who saw positive or negative framings.

### 3.3 Main Effects Choice Model

MMNL results are presented in Table 3. Participants made choices jointly based on all three attributes included (Model 1). As expected, coefficients for effectiveness, monthly and three‐monthly compared to daily, and non‐hormonal compared to hormonal were all positive and significant at the 1% level. Marginal rate of substitution results show that respondents were willing to trade effectiveness for other desirable attributes. For example, respondents were willing to trade 3.6% points of yearly efficacy for a non‐hormonal contraceptive compared to a hormonal method. Participants were also willing to trade 2.3 and 2.5% points of yearly efficacy for an option that is to be taken monthly or three‐monthly compared to daily, respectively.

**TABLE 3:** Mixed multinomial logit choice models.

			(1) No framing		(2) Pos. Neg. Framing interacted with effectiveness		(3) Year framing interacted with effectiveness	
		Value	Coeff.	SE	Coeff.	SE	Coeff.	SE
Attribute								
Effectiveness		Mean	0.65***	0.05	0.72***	0.05	0.89***	0.06
		SD	0.47***	0.04	0.47***	0.04	0.33***	0.04
Frequency	Daily (ref)							
	Monthly	Mean	1.51***	0.14	1.52***	0.13	2.61***	0.21
		SD	0.31	0.29	0.17	0.38	0.67***	0.23
	Three‐monthly	Mean	1.60***	0.15	1.60**	0.15	2.98***	0.23
		SD	0.68***	0.25	0.7***	0.24	0.26	0.47
Type	Hormonal (ref)							
	Non‐hormonal	Mean	2.32***	0.19	2.33***	0.18	3.64***	0.28
		SD	2.11***	0.17	2.08***	0.17	2.45***	0.22
Positive and negative framing								
Effectiveness								
Pos. Neg. Framing x effectiveness		Mean			−0.13***	0.04		
		SD			0.10**	0.05		
Year framing								
Effectiveness								
Year framing x effectiveness		Mean					−0.09***	0.01
		SD					0.002	0.00
Loglikelihood			−1798.3		−1793.3		−1722.1	
AIC			3612.6		3606.6		3464.3	
BIC			3671.8		3680.5		3538.2	
*N*			752		752		752	

*Note:* Pos. Neg. framing—positive versus negative framing. Year framing—1‐year versus. 3‐year framing.

*Significant at 10%.

** Significant at 5%.

*** Significant at 1%.

### 3.4 Impact of Framing on Marginal Utilities

Strong framing effects were observed under both time and risk framings—significant interaction coefficients suggest a difference in preferences between framings, indicating that participants' choices were influenced by how information is presented.

Participants were more sensitive to effectiveness in the negative frame and gained 18% more utility from 1% point of effectiveness compared to under the positive frame (Model 2).

For the time framing, participants valued 1% extra efficacy 10% less in the 3‐year framing compared to the 1‐year framing (Model 3). For comparison, the NICE discount rate of 3.5% equates to a 9.8% discount rate over 3 years.

### 3.5 Preference Heterogeneity

There were significant differences in valuation of effectiveness by participant characteristics (Supporting Inormation S1: Appendix I, Tables 2 and 3). Those who were currently using hormonal contraception valued effectiveness nearly twice as much as those who were using non‐hormonal methods (increase in utility provided by 1% point of effectiveness: 98%, *p* < 0.001). Participants who stated they used contraceptives to avoid pregnancy valued effectiveness more than those who used them for other reasons only (increase of 75%, *p* < 0.001). Women who reported that they would feeling worse if they were to become pregnant valued effectiveness more than those who reported they would feel ambivalent or happy (increase of 33%, *p* = 0.001). Finally, participants who had not been pregnant value effectiveness more than those who had (increase of 34%, *p* < 0.001).

## 4 Discussion

We found that how contraceptive effectiveness information was presented affected contraceptive choices. One percent point of effectiveness was valued 18% more in negatively framed tasks (“X% will become pregnant”) compared to a positive frame (“[1‐X%] will not become pregnant”), in line with prospect theory (Kahneman and Tversky 1979) and previous empirical literature outside of contraceptive choices (Tversky and Kahneman 1991; Neuman and Neuman 2007; Veldwijk et al. 2016). Furthermore, the time framing showed that 1% point of effectiveness was valued 10% less under a 3‐year framing than 1‐year, in line with previous literature (Shaklee and Fischhoff 1990).

We found considerable preference heterogeneity across participants with effectiveness valued more among participants currently using hormonal methods, who stated avoiding pregnancy as the reason for current contraceptive use, who had not previously been pregnant, or who reported higher pregnancy aversion. There was no evidence of heterogeneity in framing impacts, though the sample size may be too small to detect variation in small framing effects.

Just as prospect theory is consistent with risk framing finding, a discounted utility perspective is consistent with the time framing finding (Frederick et al. 2002), and this study's finding of a 10% decrease in valuation of effectiveness over 3 years is remarkably similar to a 3‐year extrapolation of NICE's 3.5% discount rate (9.8%) (National Institute for Health and Care Excellence 2001). An alternative explanation is that the 3‐year risk may seem less immediately applicable to individuals; a literature review on risk attributes in DCEs found that when presented with risks not applicable to them, individuals value risks differently (Harrison et al. 2014).

There are several limitations to this study. First, because population‐level preferences were not of key interest in the study, a convenience sampling approach was used which resulted in a sample of relatively young, well‐educated contraceptive users, and results from a representative or non‐UK‐based sample, may differ. Although evidence from syntheses of risk framing experiments suggests some heterogeneity in response to framing effects by demographics, there is inconsistent evidence of systematic heterogeneity in response to framing effects: as summarized by Kühberger (Kühberger 2023) “Although significant findings are reported here and there, no consistent picture emerges […] numeracy shows only little effects in framing studies […] matters are similar with respect to effort, experience, and nationality” (Frederick et al. 2002). Nevertheless, results from this study may be context specific and insights limited in generalizability outside of this sample. In addition, inclusion criteria required participants to be currently using contraception, which meant that women who were not actively seeking to conceive but also not actively using contraception were not included. Because the aim of the experiment was to explore sensitivity to risk framings, the intention was to restrict the sample as much as possible to those choosing methods in light of this risk information, however we acknowledge a loss of generalizability in excluding this group.

Second, to prioritize the exploration of framing effects questions, the DCE tool was intentionally very simple and stylized. Attributes and levels were derived from a literature review and not expert opinion or qualitative research as is best practice for DCEs (Quaife, Terris‐Prestholt, et al. 2018), though we note that piloting supported the use of the chosen attributes and no omitted factors were identified as more important than those included. In addition, the effectiveness level range included 99%, but highly effective long‐acting reversible contraceptives such as the inter‐uterine system or hormonal coil has an efficacy of greater than 99% (nhs.uk 2017). The decision to limit effectiveness at 99% was taken to avoid potential dominance as effectiveness approached 100% and allow a simple and consistent representation of attribute levels in integer percentage terms with reasonably‐sized risk arrays. However, omitting effectiveness values of above 99% limits the extent to which we can extrapolate the impact of framing effects to highly effective, longer‐acting methods.

Third, the DCE design did not include an opt‐out alternative. Although we did not seek to estimate unconditional demand, the lack of opt‐out may have been an issue as 31% of the sample reported current use of long‐active reversible contraception, which have use frequencies of longer than the maximum three‐monthly level included in the DCE. Fourth, because of survey software limitations, randomization was implemented based on participant odd or even integer ages. It is possible that, given the narrow band of potential ages, clusters of even‐ or odd‐aged subjects will be part of the same birth cohort and could have preferences shaped by the idiosyncrasies of that birth cohort. A model including birth‐year fixed effects would have accounted for this potential heterogeneity, however, was not possible as we only collected integer ages, not birth year data.

This study is timely because, in a context where contraceptive information is increasingly provided to potential users outside of healthcare settings, it is critical to understand how the presentation of this information can encourage optimal decision making in the absence of clinical advice. This is also important because access to safe and effective reproductive healthcare is socioeconomically patterned (Metcalfe et al. 2016; Psaki et al. 2019; Iseyemi et al. 2017)—if behavioral responses to risk information are correlated with the same characteristics, for example numeracy education, inequalities in outcomes by socioeconomic status may be exacerbated further. This study supports the need for formal guidance to standardize contraceptive effectiveness information for use in non‐clinical settings, based on robust evidence of how people respond to such information. This study also emphasizes the need for DCE studies to report design decisions around risk presentation transparently and robustly, since these decisions may ultimately affect results. Finally, these findings may be relevant in a clinical setting, as if providers are seeking to use these findings to maximize potential users' understanding and valuation of contraceptive effectiveness, they should describe effectiveness with a negative frame over a 1‐year period (i.e. “X% of women will become pregnant every year”).

## Conflicts of Interest

MQ is employed by Evidera Ltd., which provides for‐profit preference research consultancy services to pharmaceutical clients. The work supporting this manuscript was unfunded.

## Supporting information


Supporting information S1

## Data Availability

The data that support the findings of this study are available from the corresponding author upon reasonable request.
