# Supplementary material for: You've Been Framed: The Impact of Risk and Time Framings on Contraceptive Preferences in a Discrete Choice Experiment
Source: Health Econ. 2025 Sep 17;35(1):3–11. doi: 10.1002/hec.70039 (PMC12680911; doi:10.1002/hec.70039)
Supplement: Supplementary file 1 — Supporting information S1 [file HEC-35-3-s001.docx]

Appendices to: You’ve Been Framed: The impact of risk and time framings on contraceptive preferences in a discrete choice experiment

Appendix I: Supplementary Results Tables

Appendix Table 1: Direct ranking of attribute importance

| **Attribute** | **Rank** | **Total sample (N=752)** | **Positive frame (N=411)** | **Negative frame (N=341)** | **Difference between framings** $\boldsymbol{\chi}^{\boldsymbol{2}}$ **test p-value** |
| --- | --- | --- | --- | --- | --- |
| **Effectiveness** N (%) | 1st | 515 (68.48) | 280 (68.13) | 235 (68.91) | 0.93 |
|  | 2nd | 163 (21.68) | 89 (21.65) | 74 (21.70) |  |
|  | 3rd | 74 (9.84) | 42 (10.22) | 32 (9.38) |  |
| **Type** N (%) | 1st | 174 (23.14) | 95 (23.11) | 79 (23.17) | 0.11 |
|  | 2nd | 343 (45.61) | 200 (48.66) | 143 (41.94) |  |
|  | 3rd | 235 (31.25) | 116 (28.22) | 119 (34.90) |  |
| **Frequency**  N (%) | 1st | 63 (8.38) | 36 (8.76) | 27 (7.92) | 0.15 |
|  | 2nd | 246 (32.71) | 122 (29.68) | 124 (36.36) |  |
|  | 3rd | 443 (58.91) | 253 (61.56) | 190 (55.72) |  |

Appendix Table 2: MMNL Models with Participant Characteristics

| **Respondent characteristic models** | | **Value** | **Model 2A** | | **Log likelihood** | **AIC** | **BIC** | **N** |
| --- | --- | --- | --- | --- | --- | --- | --- | --- |
|  |  |  | **Coeff.** | **SE** |  |  |  |  |
| **Age** (27 years and above) | Below 27yrs (ref) |  |  |  | -1794.1 | 3609.9 | 3683.9 | 752 |
|  | Above 27 years x effectiveness | Mean | -0.06 | 0.06 |  |  |  |  |
|  |  | SD | 0.18* | 0.1 |  |  |  |  |
|  | Effectiveness | Mean | 0.68*** | 0.05 |  |  |  |  |
|  |  | SD | 0.47*** | 0.1 |  |  |  |  |
|  | Daily (ref) |  |  |  |  |  |  |  |
|  | Monthly | Mean | 1.54*** | 0.14 |  |  |  |  |
|  |  | SD | 0.37 | 0.26 |  |  |  |  |
|  | Three-monthly | Mean | 1.63*** | 0.15 |  |  |  |  |
|  |  | SD | 0.71*** | 0.25 |  |  |  |  |
|  | Hormonal (ref) |  |  |  |  |  |  |  |
|  | Non-hormonal | Mean | 2.37*** | 0.20 |  |  |  |  |
|  |  | SD | 2.13*** | 0.18 |  |  |  |  |
| **Current BC Used** (hormonal) | Non-hormonal BC used (ref) |  |  |  | -1761.0 | 3542.0 | 3616.0 | 752 |
|  | Hormonal BC used x effectiveness | Mean | 0.41*** | 0.05 |  |  |  |  |
|  |  | SD | 0.35*** | 0.05 |  |  |  |  |
|  | Effectiveness | Mean | 0.42*** | 0.03 |  |  |  |  |
|  |  | SD | 0.36*** | 0.03 |  |  |  |  |
|  | Daily (ref) |  |  |  |  |  |  |  |
|  | Monthly | Mean | 1.46*** | 0.12 |  |  |  |  |
|  |  | SD | 0.09 | 0.38 |  |  |  |  |
|  | Three-monthly | Mean | 1.53*** | 0.13 |  |  |  |  |
|  |  | SD | 0.62*** | 0.22 |  |  |  |  |
|  | Hormonal (ref) |  |  |  |  |  |  |  |
|  | Non-hormonal | Mean | 2.25*** | 0.17 |  |  |  |  |
|  |  | SD | 2.03*** | 0.15 |  |  |  |  |
| **Education** (university degree and above) | Below university (ref) |  |  |  | -1782.8 | 3585.5 | 3659.4 | 752 |
|  | University or above x effectiveness | Mean | 0.07 | 0.05 |  |  |  |  |
|  |  | SD | 0.33*** | 0.04 |  |  |  |  |
|  | Effectiveness | Mean | 0.61*** | 0.07 |  |  |  |  |
|  |  | SD | 0.39*** | 0.04 |  |  |  |  |
|  | Daily (ref) |  |  |  |  |  |  |  |
|  | Monthly | Mean | 1.52*** | 0.14 |  |  |  |  |
|  |  | SD | 0.33 | 0.30 |  |  |  |  |
|  | Three-monthly | Mean | 1.62*** | 0.16 |  |  |  |  |
|  |  | SD | 0.69*** | 0.26 |  |  |  |  |
|  | Hormonal (ref) |  |  |  |  |  |  |  |
|  | Non-hormonal | Mean | 2.35*** | 0.20 |  |  |  |  |
|  |  | SD | 2.10*** | 0.17 |  |  |  |  |
| **Risk Aversion score** (more risk averse) | Less risk averse (ref) |  |  |  | -1796.2 | 3612.4 | 3686.3 | 752 |
|  | More risk averse x effectiveness | Mean | 0.03 | 0.04 |  |  |  |  |
|  |  | SD | 0.26*** | 0.05 |  |  |  |  |
|  | Effectiveness | Mean | 0.64*** | 0.05 |  |  |  |  |
|  |  | SD | 0.44*** | 0.04 |  |  |  |  |
|  | Daily (ref) |  |  |  |  |  |  |  |
|  | Monthly | Mean | 1.51*** | 0.13 |  |  |  |  |
|  |  | SD | 0.21 | 0.31 |  |  |  |  |
|  | Three-monthly | Mean | 1.60*** | 0.15 |  |  |  |  |
|  |  | SD | 0.67*** | 0.25 |  |  |  |  |
|  | Hormonal (ref) |  |  |  |  |  |  |  |
|  | Non-hormonal | Mean | 2.34*** | 0.19 |  |  |  |  |
|  |  | SD | 2.07*** | 0.16 |  |  |  |  |
| **Reason for Taking BC (**to avoid pregnancy) | Not for pregnancy (ref) |  |  |  | -1790.9 | 3601.8 | 3675.7 | 752 |
|  | To avoid pregnancy x effectiveness | Mean | 0.28*** | 0.08 |  |  |  |  |
|  |  | SD | 0.24*** | 0.04 |  |  |  |  |
|  | Effectiveness | Mean | 0.38*** | 0.08 |  |  |  |  |
|  |  | SD | 0.41*** | 0.04 |  |  |  |  |
|  | Daily (ref) |  |  |  |  |  |  |  |
|  | Monthly | Mean | 1.50*** | 0.13 |  |  |  |  |
|  |  | SD | 0.13 | 0.38 |  |  |  |  |
|  | Three-monthly | Mean | 1.59*** | 0.14 |  |  |  |  |
|  |  | SD | 0.67*** | 0.23 |  |  |  |  |
|  | Hormonal (ref) |  |  |  |  |  |  |  |
|  | Non-hormonal | Mean | 2.32*** | 0.17 |  |  |  |  |
|  |  | SD | 2.08*** | 0.16 |  |  |  |  |
| **Previous pregnancy^†^**  (has been pregnant) | Not been pregnant (ref) |  |  |  | -1779.3 | 3578.6 | 3652.5 | 747 |
|  | Previously pregnant x effectiveness | Mean | -0.23*** | 0.05 |  |  |  |  |
|  |  | SD | 0.05 | 0.10 |  |  |  |  |
|  | Effectiveness | Mean | 0.67*** | 0.04 |  |  |  |  |
|  |  | SD | 0.44*** | 0.10 |  |  |  |  |
|  | Daily (ref) |  |  |  |  |  |  |  |
|  | Monthly | Mean | 1.46*** | 0.12 |  |  |  |  |
|  |  | SD | 0.01 | 0.64 |  |  |  |  |
|  | Three-monthly | Mean | 1.55*** | 0.13 |  |  |  |  |
|  |  | SD | 0.53* | 0.29 |  |  |  |  |
|  | Hormonal (ref) |  |  |  |  |  |  |  |
|  | Non-hormonal | Mean | 2.22*** | 0.16 |  |  |  |  |
|  |  | SD | 2.05*** | 0.15 |  |  |  |  |
| **Pregnancy intention^†^**  (did not intend) | Planned or unsure (ref) |  |  |  | -406.9 | 833.7 | 891.0 | 143 |
|  | Unintended pregnancy x effectiveness | Mean | 0.51 | 0.11 |  |  |  |  |
|  |  | SD | 0.10 | 0.09 |  |  |  |  |
|  | Effectiveness | Mean | 0.49*** | 0.11 |  |  |  |  |
|  |  | SD | 0.47*** | 0.10 |  |  |  |  |
|  | Daily (ref) |  |  |  |  |  |  |  |
|  | Monthly | Mean | 1.90*** | 0.41 |  |  |  |  |
|  |  | SD | 0.62 | 0.58 |  |  |  |  |
|  | Three-monthly | Mean | 1.69*** | 0.40 |  |  |  |  |
|  |  | SD | 1.39** | 0.60 |  |  |  |  |
|  | Hormonal (ref) |  |  |  |  |  |  |  |
|  | Non-hormonal | Mean | 2.99*** | 0.59 |  |  |  |  |
|  |  | SD | 2.55*** | 0.50 |  |  |  |  |
| **Pregnancy Aversion score** (more pregnancy averse) | Less pregnancy averse (ref) |  |  |  | -1792.0 | 3604.0 | 3678.0 | 752 |
|  | More pregnancy averse x effectiveness | Mean | 0.18*** | 0.05 |  |  |  |  |
|  |  | SD | 0.30*** | 0.05 |  |  |  |  |
|  | Effectiveness | Mean | 0.53*** | 0.04 |  |  |  |  |
|  |  | SD | 0.41*** | 0.04 |  |  |  |  |
|  | Daily (ref) |  |  |  |  |  |  |  |
|  | Monthly | Mean | 1.47*** | 0.12 |  |  |  |  |
|  |  | SD | 0.05 | 0.35 |  |  |  |  |
|  | Three-monthly | Mean | 1.55*** | 0.13 |  |  |  |  |
|  |  | SD | 0.59** | 0.24 |  |  |  |  |
|  | Hormonal (ref) |  |  |  |  |  |  |  |
|  | Non-hormonal | Mean | 2.28*** | 0.17 |  |  |  |  |
|  |  | SD | 2.04*** | 0.15 |  |  |  |  |

*** - Significant at 1%. ** - Significant at 5%. * - Significant at 10%

**^†^ -** “Prefer not to answer” responses removed from analysis

Appendix Table 3: MMNL Models with Risk and Time Framings

| **Respondent characteristics and year frame models** | | **Value** | **Model 5C** | | **Log likelihood** | **AIC** | **BIC** | **N** |
| --- | --- | --- | --- | --- | --- | --- | --- | --- |
|  |  |  | **Coeff.** | **SE** |  |  |  |  |
| **Age** (27 yrs and above) | Below 27yrs (ref) |  |  |  | -1721.3 | 3470.5 | 3574.1 | 752 |
|  | Above 27 years x effectiveness x year framing | Mean | -0.003 | 0.01 |  |  |  |  |
|  |  | SD | 0.003 | 0.01 |  |  |  |  |
|  | Above 27 years x effectiveness | Mean | -0.04 | 0.04 |  |  |  |  |
|  |  | SD | 0.06 | 0.04 |  |  |  |  |
|  | Effectiveness x year framing | Mean | -0.08*** | 0.01 |  |  |  |  |
|  |  | SD | 0.000 | 0.01 |  |  |  |  |
|  | Effectiveness | Mean | 0.89*** | 0.06 |  |  |  |  |
|  |  | SD | 0.31*** | 0.03 |  |  |  |  |
|  | Daily (ref) |  |  |  |  |  |  |  |
|  | Monthly | Mean | 2.59*** | 0.19 |  |  |  |  |
|  |  | SD | 0.65*** | 0.22 |  |  |  |  |
|  | Three-monthly | Mean | 2.96*** | 0.21 |  |  |  |  |
|  |  | SD | 0.01 | 0.66 |  |  |  |  |
|  | Hormonal (ref) |  |  |  |  |  |  |  |
|  | Non-hormonal | Mean | 3.60*** | 0.25 |  |  |  |  |
|  |  | SD | 2.42*** | 0.18 |  |  |  |  |
| **Current BC Used** (hormonal) | Non-hormonal BC used (ref) |  |  |  | -1681.5 | 3391.0 | 3494.6 | 752 |
|  | Hormonal BC used x effectiveness x year framing | Mean | 0.001 | 0.01 |  |  |  |  |
|  |  | SD | 0.02*** | 0.005 |  |  |  |  |
|  | Hormonal BC used x effectiveness | Mean | 0.39*** | 0.06 |  |  |  |  |
|  |  | SD | 0.31*** | 0.04 |  |  |  |  |
|  | Effectiveness x year framing | Mean | -0.08*** | 0.01 |  |  |  |  |
|  |  | SD | <0.001 | 0.005 |  |  |  |  |
|  | Effectiveness | Mean | 0.74*** | 0.06 |  |  |  |  |
|  |  | SD | 0.27*** | 0.03 |  |  |  |  |
|  | Daily (ref) |  |  |  |  |  |  |  |
|  | Monthly | Mean | 2.69*** | 0.23 |  |  |  |  |
|  |  | SD | 0.79*** | 0.22 |  |  |  |  |
|  | Three-monthly | Mean | 3.05*** | 0.25 |  |  |  |  |
|  |  | SD | 0.50 | 0.30 |  |  |  |  |
|  | Hormonal (ref) |  |  |  |  |  |  |  |
|  | Non-hormonal | Mean | 3.79*** | 0.25 |  |  |  |  |
|  |  | SD | 2.52*** | 0.23 |  |  |  |  |
| **Education** (university degree and above) | Below university (ref) |  |  |  | -1706.7 | 3441.3 | 3544.8 | 752 |
|  | University or above x effectiveness x year framing | Mean | 0.01 | 0.01 |  |  |  |  |
|  |  | SD | 0.007 | 0.01 |  |  |  |  |
|  | University or above x effectiveness | Mean | 0.04 | 0.05 |  |  |  |  |
|  |  | SD | 0.28*** | 0.04 |  |  |  |  |
|  | Effectiveness x year framing | Mean | -0.10*** | 0.01 |  |  |  |  |
|  |  | SD | 0.002 | 0.01 |  |  |  |  |
|  | Effectiveness | Mean | 0.86*** | 0.07 |  |  |  |  |
|  |  | SD | 0.23*** | 0.04 |  |  |  |  |
|  | Daily (ref) |  |  |  |  |  |  |  |
|  | Monthly | Mean | 2.61*** | 0.23 |  |  |  |  |
|  |  | SD | 0.64** | 0.23 |  |  |  |  |
|  | Three-monthly | Mean | 2.99*** | 0.26 |  |  |  |  |
|  |  | SD | 0.34 | 0.32 |  |  |  |  |
|  | Hormonal (ref) |  |  |  |  |  |  |  |
|  | Non-hormonal | Mean | 3.64*** | 0.30 |  |  |  |  |
|  |  | SD | 2.44*** | 0.24 |  |  |  |  |
| **Risk Aversion score** (more risk averse) | Less risk averse (ref) |  |  |  | -1722.0 | 3472.0 | 3575.5 | 752 |
|  | More risk averse x effectiveness x year framing | Mean | <0.001 | 0.008 |  |  |  |  |
|  |  | SD | 0.006 | 0.008 |  |  |  |  |
|  | More risk averse x effectiveness | Mean | 0.03 | 0.04 |  |  |  |  |
|  |  | SD | 0.18*** | 0.05 |  |  |  |  |
|  | Effectiveness x year framing | Mean | -0.08*** | 0.01 |  |  |  |  |
|  |  | SD | 0.002 | 0.01 |  |  |  |  |
|  | Effectiveness | Mean | 0.86*** | 0.06 |  |  |  |  |
|  |  | SD | 0.31*** | 0.03 |  |  |  |  |
|  | Daily (ref) |  |  |  |  |  |  |  |
|  | Monthly | Mean | 2.54*** | 0.20 |  |  |  |  |
|  |  | SD | 0.54** | 0.30 |  |  |  |  |
|  | Three-monthly | Mean | 2.90*** | 0.20 |  |  |  |  |
|  |  | SD | 0.17 | 0.52 |  |  |  |  |
|  | Hormonal (ref) |  |  |  |  |  |  |  |
|  | Non-hormonal | Mean | 3.55*** | 0.27 |  |  |  |  |
|  |  | SD | 2.40*** | 0.20 |  |  |  |  |
| **Reason for Taking BC (**to avoid pregnancy) | Not for pregnancy (ref) |  |  |  | -1713.9 | 3455.9 | 3559.4 | 752 |
|  | To avoid pregnancy x effectiveness x year framing | Mean | -0.002 | 0.01 |  |  |  |  |
|  |  | SD | 0.005 | 0.005 |  |  |  |  |
|  | To avoid pregnancy x effectiveness | Mean | 0.26*** | 0.08 |  |  |  |  |
|  |  | SD | 0.19*** | 0.04 |  |  |  |  |
|  | Effectiveness x year framing | Mean | -0.08*** | 0.01 |  |  |  |  |
|  |  | SD | <0.001 | 0.01 |  |  |  |  |
|  | Effectiveness | Mean | 0.65*** | 0.08 |  |  |  |  |
|  |  | SD | 0.27*** | 0.03 |  |  |  |  |
|  | Daily (ref) |  |  |  |  |  |  |  |
|  | Monthly | Mean | 2.60*** | 0.20 |  |  |  |  |
|  |  | SD | 0.66*** | 0.23 |  |  |  |  |
|  | Three-monthly | Mean | 2.98*** | 0.23 |  |  |  |  |
|  |  | SD | 0.21 | 0.52 |  |  |  |  |
|  | Hormonal (ref) |  |  |  |  |  |  |  |
|  | Non-hormonal | Mean | 3.64*** | 0.28 |  |  |  |  |
|  |  | SD | 2.46*** | 0.21 |  |  |  |  |
| **Previous pregnancy^†^**  (has been pregnant) | Not been pregnant (ref) |  |  |  | -1697.0 | 3422.1 | 3535.5 | 747 |
|  | Previously pregnant x effectiveness x year framing | Mean | 0.02*** | 0.01 |  |  |  |  |
|  |  | SD | 0.01 | 0.01 |  |  |  |  |
|  | Previously pregnant x effectiveness | Mean | -0.26*** | 0.05 |  |  |  |  |
|  |  | SD | 0.04 | 0.06 |  |  |  |  |
|  | Effectiveness x year framing | Mean | -0.09*** | 0.01 |  |  |  |  |
|  |  | SD | 0.003 | 0.01 |  |  |  |  |
|  | Effectiveness | Mean | 0.97*** | 0.07 |  |  |  |  |
|  |  | SD | 0.33*** | 0.03 |  |  |  |  |
|  | Daily (ref) |  |  |  |  |  |  |  |
|  | Monthly | Mean | 2.69*** | 0.22 |  |  |  |  |
|  |  | SD | 0.76*** | 0.22 |  |  |  |  |
|  | Three-monthly | Mean | 3.07*** | 0.25 |  |  |  |  |
|  |  | SD | 0.42 | 0.35 |  |  |  |  |
|  | Hormonal (ref) |  |  |  |  |  |  |  |
|  | Non-hormonal | Mean | 3.74*** | 0.30 |  |  |  |  |
|  |  | SD | 2.52*** | 0.23 |  |  |  |  |
| **Pregnancy intention^†^**  (did not intend) | Planned or unsure (ref) |  |  |  | -393.8 | 815.7 | 896.0 | 143 |
|  | Unintended pregnancy x effectiveness x year framing | Mean | 0.03* | 0.02 |  |  |  |  |
|  |  | SD | 0.003 | 0.01 |  |  |  |  |
|  | Unintended pregnancy x effectiveness | Mean | -0.07 | 0.09 |  |  |  |  |
|  |  | SD | 0.03 | 0.07 |  |  |  |  |
|  | Effectiveness x year framing | Mean | -0.08*** | 0.02 |  |  |  |  |
|  |  | SD | 0.01 | 0.01 |  |  |  |  |
|  | Effectiveness | Mean | 0.76*** | 0.13 |  |  |  |  |
|  |  | SD | 0.36*** | 0.07 |  |  |  |  |
|  | Daily (ref) |  |  |  |  |  |  |  |
|  | Monthly | Mean | 2.72*** | 0.48 |  |  |  |  |
|  |  | SD | 0.96** | 0.44 |  |  |  |  |
|  | Three-monthly | Mean | 2.77*** | 0.51 |  |  |  |  |
|  |  | SD | 1.15** | 0.46 |  |  |  |  |
|  | Hormonal (ref) |  |  |  |  |  |  |  |
|  | Non-hormonal | Mean | 3.98*** | 0.65 |  |  |  |  |
|  |  | SD | 2.95*** | 0.57 |  |  |  |  |
| **Pregnancy Aversion score** (more pregnancy averse) | Less pregnancy averse (ref) |  |  |  | -1715.7 | 3459.3 | 3562.9 | 752 |
|  | More pregnancy averse x effectiveness x year framing | Mean | -0.004 | 0.01 |  |  |  |  |
|  |  | SD | 0.01 | 0.01 |  |  |  |  |
|  | More pregnancy averse x effectiveness | Mean | 0.16*** | 0.04 |  |  |  |  |
|  |  | SD | 0.19*** | 0.04 |  |  |  |  |
|  | Effectiveness x year framing | Mean | -0.08*** | 0.01 |  |  |  |  |
|  |  | SD | <0.001 | 0.01 |  |  |  |  |
|  | Effectiveness | Mean | 0.79*** | 0.05 |  |  |  |  |
|  |  | SD | 0.30*** | 0.03 |  |  |  |  |
|  | Daily (ref) |  |  |  |  |  |  |  |
|  | Monthly | Mean | 2.56*** | 0.19 |  |  |  |  |
|  |  | SD | 0.56** | 0.26 |  |  |  |  |
|  | Three-monthly | Mean | 2.92*** | 0.22 |  |  |  |  |
|  |  | SD | 0.33 | 0.30 |  |  |  |  |
|  | Hormonal (ref) |  |  |  |  |  |  |  |
|  | Non-hormonal | Mean | 3.56*** | 0.25 |  |  |  |  |
|  |  | SD | 2.44*** | 0.20 |  |  |  |  |

*** - Significant at 1%. ** - Significant at 5%. * - Significant at 10%

Year framing – one-year vs three-year framing **^†^ -** “Prefer not to answer” responses removed from analysis
